# MALDI-TOF质谱筛查NSCLC患者血清特异性多肽的探索性研究

**DOI:** 10.3779/j.issn.1009-3419.2013.05.04

**Published:** 2013-05-20

**Authors:** 娟 安, 传昊 汤, 娜 王, 毅 刘, 万峰 郭, 晓燕 李, 子赫 王, 昆 何, 晓晴 刘

**Affiliations:** 1 100071 北京，军事医学科学院附属医院肺部肿瘤科 Department of Lung Cancer, Affiliated Hospital of Academy of Military Medical Sciences, Beijing 100071, China; 2 100850 北京，国家生物医学分析中心 National Center of Biomedical Analysis, Beijing 100850, China; 3 100071 北京，军事医学科学院附属医院肿瘤学研究室 Laboratory of Oncology, Affiliated Hospital of Academy of Military Medical Sciences, Beijing 100071, China

**Keywords:** 铜离子鳌合纳米磁珠, 基质辅助激光解析电离飞行时间质谱, 肿瘤蛋白质组学, 血清多肽图, 肺肿瘤, Magnetic beads- immobilized metal affinity capture - copper, Matrix-assisted laser desorption/ionization time-of-flight mass spectrometry, Cancer proteomics, Serum peptide profiles, Lung neoplasms

## Abstract

**背景与目的:**

早期诊断是提高肺癌生存率的关键，传统的肺癌诊断技术仍存在一定局限性。鉴于近年来以质谱为核心技术的肿瘤蛋白组学在癌症诊断方面的初步研究，本研究探索性应用基质辅助激光解析电离飞行时间质谱（matrix assisted laser desorption ionization-time of flight-mass spectrometry, MALDI-TOF-MS）分析非小细胞肺癌（non-small cell lung cancer, NSCLC）患者和健康人群的血清差异多肽，以建立NSCLC的血清分类模型。

**方法:**

将年龄和性别匹配的133例NSCLC患者和132例健康者血清标本按照3:1的比例随机分为两组：训练组由100例NSCLC患者和100例健康者血清标本组成，用以建立分类模型；测试组由33例NSCLC患者和32例健康者血清标本组成，用以验证模型。采用铜离子鳌合纳米磁珠提取血清多肽、MALDI-TOF-MS技术检测得到质谱图。ClinProTools^TM^统计软件分析训练组NSCLC患者与健康者之间的多肽图谱，从中筛选出一组差异多肽并建立分类模型，最后用测试组对模型进行盲样验证。

**结果:**

在训练组中观察到血清质荷比（m/z）在1, 000 Da-10, 000 Da范围内有131个差异多肽信号峰，在此范围内共得到14个有统计学意义的差异多肽峰（*P* < 0.000, 001; AUC≥0.9），其中NSCLC患者与健康者相比，表达上调的多肽有2个，表达下调的有12个，由统计软件筛选出3个多肽峰（7, 478.59 Da、2, 271.44 Da、4, 468.38 Da）建立分类模型，然后对测试组进行验证，其盲样验证敏感性100%，特异性96.9%，准确率98.5%。

**结论:**

本组研究显示NSCLC患者与健康人群的血清多肽存在差异，应用MALDI-TOF-MS技术可建立NSCLC的血清多肽分类模型且小规模验证具有较好的敏感性和特异性，希望大规模验证模型，并与传统诊断方法对照或结合，进而尝试建立一种新的NSCLC早期诊断模式。

## Abstract (en)

**Background and objective:**

The improved survival of patients with lung cancer depends on early diagnosis of lung cancer. However, the traditional diagnostic techniques have several limitations. Mass spectrometry (MS) has been applied as a core technology for cancer diagnosis in preliminary proteomic studies. The aim of this study is to explore the differences in the serum peptide levels of patients with non-small cell lung cancer (NSCLC) and healthy individuals using matrix-assisted laser desorption/ionization (MALDI)-time-of-flight (TOF)-MS. A NSCLC serum classification model was then established.

**Methods:**

One hundred and thirty three cases of patients with NSCLC serum specimens and 132 cases of healthy human serum specimens were randomly divided into two groups in accordance with the ratio of three to one without age and gender differences. The training group was used to establish the classification model, this group included serum samples from 100 NSCLC cases and 100 healthy individuals. The test group for validating the proposed model was composed of the remaining serum samples from 33 NSCLC cases and 32 healthy individuals. Peptides were extracted from the samples using magnetic beads- immobilized metal affinity capture - copper, and their mass spectra were obtained using an automated MALDI-TOF-MS system. The MS data from the training group was analyzed using the ClinproTool^TM^ software to identify the individual peptide fragments and establish the classification model. The sensitivity and specificity of the model were verified by blind testing with the test group.

**Results:**

Among the 131 different peptide peaks, ranging from m/z 1, 000 Da to 10, 000 Da, 14 peaks were significantly different in the NSCLC samples of the training group, as compared with the controls (*P* < 0.000, 001; AUC≥0.9); these included 2 higher peaks and 12 lower peaks. The classification model was established, and the test group was verified for only 3 peptide peaks (7, 478.59, 2, 271.44 and 4, 468.38 Da), which were selected by the statistical software. Blind testing revealed that the proposed method had 100% sensitivity, 96.9% specificity and 98.5% accuracy.

**Conclusion:**

Our results showed that the serum peptide levels were significantly different between NSCLC patients and healthy individuals. A serum peptide-based classification of NSCLC patients was established using an automated MALDI-TOF-MS system. This method demonstrated high sensitivity and specificity in a small-scale test. Future studies should test the proposed model through mass validation. The model could be compared or combined with traditional diagnostic methods to establish novel techniques for the early diagnosis of patients with NSCLC.

肺癌是常见的恶性肿瘤之一，近年来发病率上升速度高居各种肿瘤之首。据卫生部2008年统计数据显示，我国每年肺癌的发病人数约为70万，2/3的患者发现时已失去手术根治机会。其中，占肺癌80%-90%的非小细胞肺癌（non-small cell lung cancer, NSCLC）患者5年生存率仅为8%-15%^[1]^，但Ⅰ期肺癌术后10年生存率可达到92%^[2]^。由此可见早诊断早治疗是降低肺癌死亡率的关键所在。传统的医学诊断主要通过影像学检查，尽管低剂量螺旋CT的应用相比胸部X线片提高了肺癌早诊率，死亡率下降了20%^[3]^，然而，早期肺癌患者体内潜在癌变的细胞克隆远远小于现今影像学测量技术可达的最小阈值，因此有必要寻找更早于影像学诊断的灵敏性和特异性更高的方法以提高肺癌的早期确诊率。肿瘤蛋白质组学从蛋白、肽段等更微观的角度研究肿瘤，寻找肿瘤标志蛋白，进而阐明其表达水平的变化与肿瘤发生、发展的相互关系，在肿瘤早期诊断中极具潜力。其核心技术质谱分析近年已逐渐应用于乳腺癌、胃癌、大肠癌等恶性肿瘤早期诊断的探索性研究^[4-6]^。本研究旨在应用基质辅助激光解析电离飞行时间质谱（matrix assisted laser desorption ionization-time of flight-mass spectrometry, MALDI-TOF-MS）与纳米磁珠提取血清多肽方法相结合--ClinProt^TM^系统，探索性分析NSCLC患者与健康人群的血清，从中筛选差异性多肽，通过统计学方法建立诊断NSCLC的分类模型并进行验证，以期为建立NSCLC血清学诊断方法奠定基础。

## 1 材料与方法

### 1.1 材料

#### 1.1.1 标本来源与收集

共收集了2011年8月-2012年10月在军事医学科学院附属医院肺部肿瘤科就诊，经病理证实为NSCLC的患者的血清标本（以下简称肺癌组）133例，所有病例均经临床检查排除重要脏器（心、肝、肾等）疾病，血清标本在患者未进行任何治疗时采集。同期收集了来自门诊体检正常的健康者的血清标本（以下简称健康组）132例，所有健康者肺部专科检查无异常，既往无肺部疾病病史。

标本收集步骤：①晨起8:00空腹采集外周静脉全血3 mL置于EDTA抗凝试管；②常温静置2 h；③于4 ℃、1, 500 r/min离心10 min；④取上清液（血清），每150 μL分装一份，-80 ℃冰箱保存。

#### 1.1.2 入组者基本情况

将133例NSCLC患者和132例健康者血清标本分别按3:1的比例随机分为两组：训练组由100例NSCLC患者（以下简称肺癌组Ⅰ）和100例健康者（以下简称健康组Ⅰ）血清标本组成，用以建立分类模型；测试组由33例NSCLC患者（以下简称肺癌组Ⅱ）和32例健康者（以下简称健康组Ⅱ）血清标本组成，用以验证模型。训练组与测试组患者在年龄、性别、吸烟情况、组织学类型及临床分期等方面均无统计学差异。患者及健康者基本情况见表 1。

**1:** 患者及健康者基本情况 Basic information of patients and healthy human

Characteristic	Training group (*n*=200)				Test group (*n*=65)		
	NSCLC Ⅰ (*n*=100)	Healthy Ⅰ (*n*=100)	*P*		NSCLC Ⅱ (*n*=33)	Healthy Ⅱ (*n*=32)	*P*
Age (year)			0.849				0.673
Median	59	59			57	59	
Range	36-83	35-85			33-77	34-83	
Gender			0.774				0.916
Male	61 (61%)	59 (59%)			19 (58%)	18 (56%)	
Female	39 (39%)	41 (41%)			14 (42%)	14 (44%)	
Smoking			0.482				0.910
Yes	51 (51%)	46 (46%)			15 (45%)	15 (47%)	
No	49 (49%)	54 (54%)			18 (55%)	17 (53%)	
Pathological							
Adeno	73 (73%)	-	-		29 (88%)	-	-
Squamous	14 (14%)	-	-		2 (6%)	-	-
Else	13 (13%)	-	-		2 (6%)	-	-
Staging							
Ⅰ	1 (1%)	-	-		0	-	-
Ⅱ	0	-	-		0	-	-
Ⅲ	6 (6%)	-	-		4 (12%)	-	-
Ⅳ	93 (93%)	-	-		29 (88%)	-	-

#### 1.1.3 试剂和仪器

三氟乙酸（trifluoroacetic acid, TFA）（美国Sigma公司）；乙腈（acetonitrile，CAN，美国Fisher公司）；α-氰基-4-羟基肉桂酸（α-cyano-4-hydroxycinnamic acid, HCCA）和混合标肽（Bruker公司）；铜离子螯合纳米磁珠（MB-IMAC-Cu^2+^）及其缓冲液体系（国家生物医学分析中心）；MALDI-TOF-MS（Ultraflex）、MTP-Anchorpchip靶（Var/384）、磁珠分离器（magnetic bead separator, MBS）、分析软件ClinProTools 2.1均为Bruker公司产品；离心机（LDZ5-2，北京离心机厂）。

### 1.2 磁珠提取血清多肽及质谱检测

将MB-IMAC-Cu^2+^混匀，取5 μL磁珠及50 μL结合液加入Eppendorf管，放在MBS上移动4次，弃去上清液，并重复3次；再加入20 μL结合液和5 μL血清，混匀，室温下静置10 min，编号；将Eppendorf管放在MBS上移动4次，除去上清液；加入100 μL洗涤液，在MBS上移动4次，弃去上清液；再加入100 μL洗涤液，在MBS上移动4次，弃去上清液，此步骤重复2次；加入20 μL洗脱液，混匀，室温静置20 min，将Eppendorf管放置在MBS上移动4次后静置20 s，将上清液移至新的Eppendorf管内，得到血清多肽，编号。

将1 μL提取的血清多肽与1 μL饱和HCCA基质（用含0.1%TFA、50%CAN溶解）混匀，点在靶板上，室温干燥；将靶板放入质谱仪；用标准品校正仪器后，检测标品，获得由不同质荷比（mass-to-charge ratio, m/z）的多肽峰构成的质谱图。

为了避免系统误差以及人为操作的失误，在每次标本检测前均会使用标准品（多肽混合物）进行检测，当检测结果与标准品组成一致，说明试验系统正常时才会进行标本的检测，从而保证实验结果可靠以及可重复性。

同时，为了说明系统稳定性，随机抽取肺癌组和健康组血清标本各10例，每例每一次取血清5 μL，用于连续6天以及1天内连续6次的磁珠提取和质谱实验。选取m/z范围在1, 000 Da-10, 000 Da的峰计算变异系数，结果显示日间和日内变异系数均小于20%。

### 1.3 统计学分析

血清多肽经MB-IMAC-Cu^2+^提取后，通过MALDI-TOF-MS进行线性模式检测，所获得的质谱数据通过ClinProTool^TM^（CPT）软件进行统计学分析，在用CPT软件正式分析前先对样品原始质谱图进行处理，包括基线消减、校正和归一化。随后，将作为训练组的100例NSCLC患者和100例健康者的血清多肽谱，用软件内嵌的统计算法进行统计学分析，获得两组间的差异表达多肽，然后由统计软件优选出其中3个多肽（7, 478.59 Da、2, 271.44 Da、4, 468.38 Da）建立诊断NSCLC的分类模型。模型通过分析这3个多肽在每例标本中的峰面积大小，判断该标本来源于肺癌患者或健康者。最后再用测试组的33例NSCLC患者和32例健康者通过分类模型进行盲样验证，以检验模型的稳定性和可靠性。

## 2 结果

### 2.1 训练组的血清多肽指纹图谱

训练组纳入100例NSCLC患者和100例健康者，应用MALDI-TOF-MS技术检测训练组的血清多肽磁珠提取物，得到肺癌组Ⅰ和健康组Ⅰ的血清多肽指纹图谱，结果见图 1。

**1:**
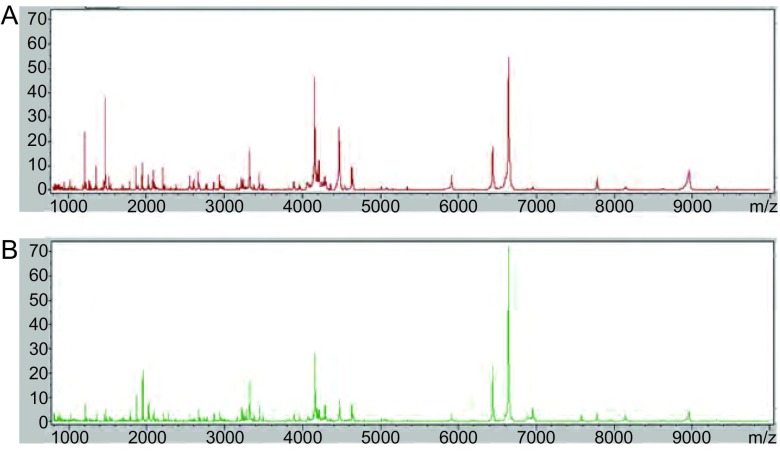
训练组的血清多肽图谱。A：肺癌组Ⅰ；B：健康组Ⅰ。 Serum peptide profiles of training group. A: NSCLC Ⅰ; B: Healthy Ⅰ.

### 2.2 训练组的血清差异多肽结果分析

#### 2.2.1 肺癌相关差异表达多肽的发现

肺癌组Ⅰ和健康组Ⅰ的血清多肽指纹图谱经CPT软件分析后共鉴定出多肽峰131个。定义两组间*P* < 0.000, 001、AUC≥0.9的多肽峰具有统计学差异，由此找到两组m/z在1, 000 Da-10, 000 Da范围内表达的差异多肽峰14个，具体结果见表 2。

**2:** 训练组差异多肽质荷比及表达变化 m/z and expression change of training group

m/z	Healthy Ⅰ (*n*=100)	NSCLC Ⅰ (*n*=100)	Expression levels of the NSCLC Ⅰ compared to Healthy Ⅰ
7, 478.59	11.55±3.78	3.85±1.42	↓
4, 468.38	150.05±51.41	434.41±157.85	↑
7, 627.28	6.86±3.37	1.73±1.02	↓
5, 992.46	4.96±1.59	2.47±1.06	↓
7, 654.04	13.91±7.69	4.25±2.07	↓
5, 290.84	11.26±6.72	2.84±1.09	↓
4, 210.42	58.23±26.12	140.56±69.97	↑
6, 973.93	36.83±26.57	6.07±5.94	↓
7, 026.53	10.72±8.07	1.69±1.01	↓
7, 944.26	26.34±19.11	5.31±3.47	↓
9, 380.50	9.67±5.83	3.20±2.22	↓
2, 271.44	37.7±23.80	12.01±4.28	↓
7, 573.48	36.77±34.02	3.05±2.09	↓
5, 043.32	20.01±12.51	6.80±9.34	↓
*P* < 0.000, 001; AUC≥0.9.			

#### 2.2.2 NSCLC分类模型的建立

通过CPT软件的聚类分析方法（图 2）对肺癌组Ⅰ与健康组Ⅰ的血清多肽指纹图谱进行对比分析，得到监督神经网络算法（supervised neural network, SNN）为最优算法，该算法是以原型为基础的分组算法，是以Kohonen’s学习型向量量化器的观点为基础，从监督相关的神经气体算法^[7]^改进而来。其工作方式为：①依据Batch-Neural-Gas算法将预先定义的原型（分组）数分布于数据中，进而根据数据密度属性建立原型的最优分布；②随后根据分组信息优化原型数在数据中的分布，同时将经验性错误最小化。该算法结果显示最优模版由3个多肽组成，其质荷比分别为7, 478.59 Da、4, 468.38 Da、2, 271.44 Da（图 3）。以该模版建立分类模型，定义当某血清多肽图中其7, 478.59 Da峰面积在（3.85±1.42）范围、4, 468.38 Da峰面积在（434.41±157.85）范围、2, 271.44 Da峰面积在（12.01±4.28）范围时，该血清来源于肺癌患者；而血清多肽图中7, 478.59 Da峰面积在（11.55±3.78）范围、4, 468.38 Da峰面积在（150.05±51.41）范围、2, 271.44 Da峰面积在（37.7±23.8）范围时，该血清来源于健康者。结果得到该模型对肺癌的识别率为98.04%，预测能力为99.07%。

**2:**
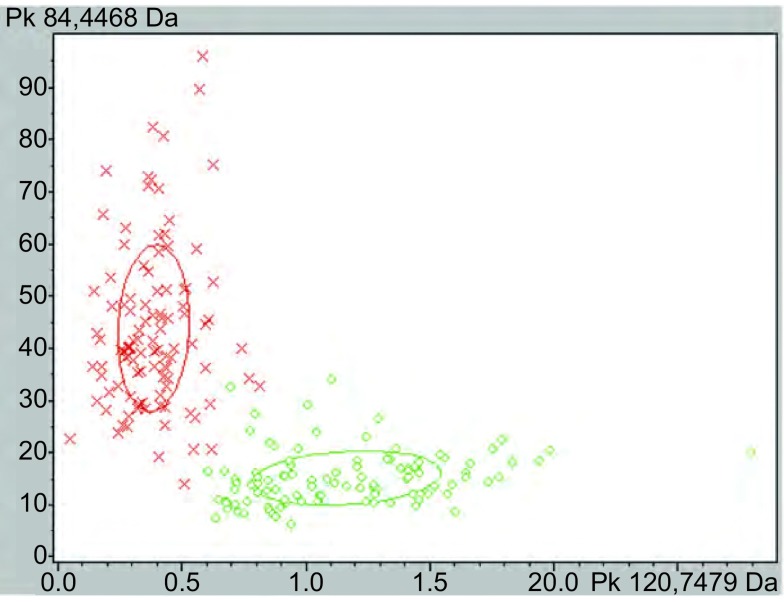
血清多肽图的聚类分析（红色：肺癌组Ⅰ；绿色：健康组Ⅰ） Clustering analysis of MS-based serum peptide profiles (red: NSCLC Ⅰ; green: Healthy Ⅰ)

**3:**
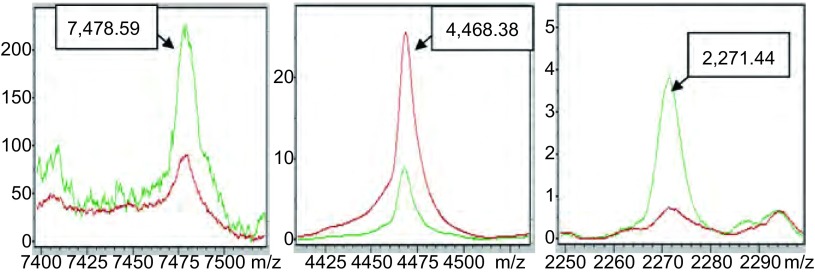
建模的3个多肽峰（红色：肺癌组Ⅰ；绿色：健康组Ⅰ） Specific peptide peaks of model (red: NSCLC Ⅰ; green: Healthy Ⅰ)

### 2.3 盲法鉴定

对纳入测试组的33例肺癌患者和32例健康者血清，采取同样的CPT软件获得其血清多肽指纹图谱，并运用SNN算法建立的模型进行验证，33例肺癌患者和31例健康对照被准确判断（其中33例肺癌患者全部判断正确，32例健康者中有1例判断假阳性，其它都判断正确）。通过盲样验证，该模型的准确率为98.5%，特异性为96.9%，敏感性为100%。

## 3 讨论

质谱分析是肿瘤蛋白质组学的核心检测技术，目前常用的生物质谱包括：MALDI-TOF-MS、电喷雾质谱（electrospray ionization-mass spectrometry, ESI-MS）、表面加强激光解析电离飞行时间质谱（surface-enhanced laser desorption ionization-time of flight-mass spectrometry, SELDI-TOF-MS）等。其中MOLID-TOF-MS具有耐盐和耐污染物的能力高、灵敏度高的特点，在仪器中引入反射技术和延迟提取技术后，仪器的分辨率大大提高，在测定2, 000 Da左右的小分子多肽时，准确度可达到10 ppm以下。

鉴于质谱在鉴定蛋白质方面具有高敏感、高通量等优势，近年来很多研究者尝试通过质谱检测来早期诊断肿瘤。其主要方法是通过对肿瘤患者与健康人群的标本进行质谱分析，从而发现肿瘤特异性蛋白或多肽。Monari等^[8]^用IMAC30-Cu和H50的SELDI芯片对44例NSCLC患者和19例正常人研究，发现两组间存在28个明显差异峰，遂建立模型，用IMAC30-Cu芯片得到的敏感性和特异性分别是70.45%和68.42%，而H50芯片分别是72.73%和73.68%。Han等^[9]^用金属亲和蛋白芯片和SELDI-TOF技术研究血清标本，利用3个蛋白质建立树状模型，盲样验证敏感性为89%、特异性为91%、准确率为90%。Hocker等^[10]^采用ESI-MS对43例早期NSCLC患者和21例对照者的血清标本进行分析，鉴定的差异蛋白区分患者及健康者血清的总体有效率和灵敏度为80%和84%。这些研究结果说明质谱检测方法有能力识别肺癌患者血清里所含有的肿瘤相关信息，有可能进行肺癌的早期诊断。但上述研究盲样验证的敏感性和特异性均较低，且没有后续研究进一步验证。分析其原因为：①在传统的方法中，无论是双向凝胶电泳、蛋白芯片还是固相萃取小柱等预处理技术提取标本中的蛋白和多肽，均存在操作复杂且提取效率较低的缺点，导致可用信息丢失；②ESI、SELDI-TOF-MS等质谱检测方法灵敏度和分辨率较低，反映的信息较少。以上限制阻碍了它们在疾病特异性标志物的发现和临床诊断上的应用。

为了解决上述问题，本研究组应用了德国布鲁克•道尔顿公司研发的ClinProt^TM^检测系统^[11, 12]^。它包括磁珠分离系统、MALDI-TOF-MS系统、分析软件和可选的体液样品自动处理系统，其优势在于：①拥有最新AnchorChip专利技术的样品靶可大大增加分析物的浓度，较常规芯片提高10倍-100倍的灵敏度；②MALDI技术的分辨率与重复性、重现性均高于SELDI或其它质谱技术；③液体磁珠总表面积大，能充分捕获血清中特异低丰度多肽信息，提高了模型的特异性和准确性；④水热法制备金属螯合纳米磁珠^[13]^系统配合了一套适合于质谱分析的缓冲溶液，整个实验体系稳定、可靠，实现了样品微量化，分析过程快速化、通量化、标准化。在哈佛大学女子医院、纽约斯隆-凯特琳癌症研究所、贝勒医学院等世界一流医院和医学研究所中，该技术已广泛应用于卵巢癌、脑胶质瘤、头颈鳞癌、前列腺癌、乳腺癌、膀胱癌^[14, 15]^等的早期诊断研究中，而在本研究中，我们通过ClinProt^TM^检测系统对NSCLC患者和健康者血清进行了检测，两组间发现14个有统计学差异的多肽峰，遂以统计软件筛选出3个多肽峰建立NSCLC分类模型，并对其进行盲样验证，其敏感性为100%，特异性为96.9%，准确率为98.5%，说明我们建立的分类模型理论上具有一定的鉴别NSCLC患者的价值，但尚需在临床实践中进一步证实。

关于我们建立的分类模型，它不像传统诊断模式那样以单一指标为判断依据，而是以一组多肽指标为判断标准。它以生物质谱技术为支撑平台，生物信息学为桥梁，对多肽表达进行研究，通过肺癌患者血液中多肽组的变化，可以发现特异的肿瘤相关信息以达到对肿瘤的诊断。当血液流经肿瘤微环境时，血清蛋白/多肽组中可能产生许多轻微的未知组分的变化，因此，使用复合的标志物可以较单个标志物更为有效。通过MALDI-TOF分析的血清质谱图需要数字化并经成熟的生物信息工具软件来诊断分析，通过与机体在正常条件下的质谱图进行比较，从而鉴别肿瘤患者和非肿瘤患者。它可在数分钟内从微量血液中测出上万个结果并对其进行分析，理论上这种分类模型在肿瘤诊断中有望超越传统的标志物检测。

本研究中建立分类模型的3个多肽峰，数据库检索显示其中m/z为2, 271.44的多肽峰可能代表间α胰蛋白酶重链H4（inter-α-trypsin inhibitor H4, ITIH4）。查阅文献，间α胰蛋白酶重链（inter-α-trypsin inhibitor, *ITIH*）基因是一种抑癌基因^[16]^，包括*ITIH1*-*ITIH5*，其中ITIH4定位于3号染色体短臂，对血浆激肽释放酶高度敏感，被认为是潜在的血浆激肽释放酶诱导的生物肽前体。它主要在炎症和癌症发生方面起重要作用^[17]^。有研究^[18]^发现ITIH4在卵巢癌血清中表达下降。也有研究^[19, 20]^认为ITIH4是乳腺癌的肿瘤标志物，在乳腺癌患者血清中表达上调，有助于诊断和判断预后。但有关肺癌的报道较少，Hamm等^[17]^发现在52%的肺癌组织中ITIH4 mRNA表达量下降。本研究结果亦显示在血清多肽图谱中该多肽峰表达下调，其在肺癌发生发展中的具体作用机制及其作为诊断标志物的价值还有待进一步研究。

本研究选用NSCLC患者以及健康者的血清作为研究对象，应用ClinProt^TM^系统的MALDI-TOF-MS技术检测血清多肽，对特异的NSCLC相关多肽小规模验证，得到了相对较高的敏感性和特异性。由于我们收集的标本主要来源于Ⅳ期患者且多为腺癌患者，以后将纳入更多的Ⅰ期患者标本甚至动态监测高危人群的标本，以及鳞癌等其它组织学类型的NSCLC标本，进一步验证该诊断模型的敏感性和特异性。用于NSCLC诊断的特异血清多肽谱模型，具有无创、标本量需求少、简便、快捷、准确性高等优势，下一步我们需要在临床实际工作中进行大规模验证，希望为建立NSCLC血清学诊断方法奠定基础。
